# The Association Between Body Mass Index, Emotional Eating and Perceived Stress during COVID-19 Partial Quarantine in Healthy Adults

**DOI:** 10.1017/S1368980021002974

**Published:** 2022-01

**Authors:** Hatice Kübra Barcın-Güzeldere, Aslı Devrim-Lanpir

**Affiliations:** 1 Faculty of Health Science, Department of Nutrition and Dietetics, Istanbul Medeniyet University, Şehit Hakan Kurban Street, Istanbul 34692, Turkey; 2 Department of Nutrition and Dietetics, Institute of Health Science, İstanbul Medipol University, Istanbul, Turkey

**Keywords:** Emotional eating, BMI, Perceived stress, COVID-19, Quarantine

## Abstract

**Objective:**

We investigated emotional eating behaviours and perceived stress during COVID-19 partial quarantine according to BMI levels in healthy adults.

**Design:**

Cross-sectional study.

**Setting:**

An online survey including demographic variables, eating attitude-related questions, Emotional Eater Questionnaire (EEQ) and Perceived Stress Scale-14 (PSS-14) was sent via online data collection platform. Self-reported weight, height and weight changes during the quarantine were also collected.

**Participants:**

A total of 506 people aged between 20–65 years who were partially quarantined due to COVID-19 participated in this study.

**Results:**

BMI was positively correlated with EEQ (*r* 0 ·205, *P* = 0·001). However, BMI was negatively linked with PSS-14 during COVID-19 (*r* -0·125, *P* = 0·001), indicating that participants with lower BMI had higher perceived stress during COVID-19. Participants gained weight during the lockdown situation (+1·20 ± 1·70 kg in men; +0·91 ± 1·40 kg in women). EEQ and PSS-14 scores of women found to be significantly higher than men (9·39 ± 5·37 in men *v*. 11·17 ± 5·85 in women for EEQ; 24·67 ± 8·32 in men *v*. 27·99 ± 7·34 in women for PSS-14). Obese participants consumed sweetened and carbonated drinks two-fold more in those compared with other participants.

**Conclusion:**

These findings suggest that partial quarantine may be closely related to emotional eating and weight gain, and participants with higher BMI showed more emotional eating behaviours. Therefore, certain precautions should be considered beforehand in order not to cause long-term eating disorder problems.

Coronaviruses (CoV) is a large family of viruses that can be cause not only mild infections seemed in common cold but also more serious infection symptoms such as Middle East Respiratory Syndrome and Severe Acute Respiratory Syndrome^(1)^. On 31 December 2019, the WHO China Country Office reported pneumonia cases of unknown aetiology in Wuhan, China’s Hubei province^(1,2)^. On 7 January 2020, the causative agent was identified as a new coronavirus (2019-nCoV) that has not previously been detected in humans. Later, the name of 2019-nCoV disease was accepted as COVID-19, and the virus was named as Severe Acute Respiratory Syndrome-CoV-2 due to its close resemblance to Severe Acute Respiratory Syndrome-CoV^(3)^. COVID-19 causes deaths on a global scale with the number of patients increasing day by day. The number of daily cases shared by the Ministry of Health in Turkey is 5·016·141 in total, 18 052 on May 8^(4)^.

The crucial and frightening feature of COVID-19 is that it can be transmitted from person to person quickly and the infection can be fatal^(5)^. Therefore, WHO has underlined that the best way to decrease the spread rate of the virus is to implement some precautions including physical and social distancing, partial or full quarantining^(6)^. Turkish Government has taken various precautions to block virus spread and reduce the number of the patients. Some of these measures in Turkey are the closure of public or crowded places such as schools, universities, cafes, gyms, mosques, quarantine for the elderly and youth, weekend quarantine for everyone and working from home^(7)^. With this pandemic, it has been reported that the time spent at home was consistently increased^(8,9)^. Parallel to the increase in the time spent at home, psychological and behavioural alterations may be observed^(8,10–14)^. Studies on COVID-19 have reported that the prevalence of anxiety disorder, depressive symptoms, perceived stress and post-traumatic stress disorder have increased with the COVID-19 pandemic^(15–20)^. In addition, studies on COVID-19 have indicated that psychological factors and increased stress due to the COVID-19 pandemic may alter eating behaviours and trigger eating disorder symptoms. Changes in eating behaviours have also been associated with increased anxiety about loss of income, restrictive orders to stay at home, fear of catching the virus and fear of losing loved ones^(21–24)^. With this in mind, the psychological changes observed during the COVID-19 pandemic may lead to eating behaviour disorders that may persist after the pandemic.

Emotional eating refers to the eating behaviour triggered by various emotions. Eating behaviours are easily affected by changes in regard to negative or positive situations^(25)^. It is well-documented that humans can consume more food than usual when they are angry or feel under pressure. Likewise, the fact that food restriction during excited or extremely stressful situations also shows the effect of emotional states on eating^(26,27)^. Studies have stated that BMI is closely associated with high stress levels and emotional eating^(28–31)^. Several studies have shown that predominantly negative emotions trigger overeating to high-fat and sugary foods, in contrary to less consumption of healthy foods, thus not complying with a healthy eating attitude (vegetables and fruits, not having breakfast, skip daily meals, more caloric food consumption)^(26,32–34)^. People struggling with emotional eating can suppress intense emotions by eating, and in this case, they often prefer appetising foods high in fat and sugar^(30,32,34,35)^. Long-term excessive consumption of foods high in fat or sugar can lead to weight gain, resulting in increased BMI and many health risks such as oxidative stress, inflammation and obesity^(36–38)^.

Stress is considered to be another key factor affecting dietary intake^(30,39)^. Stressful situations can affect diet quality scores in different ways by triggering emotional eating, increasing the consumption of high-fat and sweetened food/beverages and causing uncontrolled eating behaviour. These situations can also trigger emotional eating attacks. Studies have shown that non-obese people use coping mechanisms such as cognitive strategies to cope with psychological problems, while obese individuals may respond to these problems by overeating^(40,41)^. Thus, increased stress level can lead to weight gain, overweight and obesity, leading to an increased BMI levels^(42)^.

Perceived stress appears to be increasing during the COVID-19 pandemic, and most importantly, it seems that perceived stress may be associated with changes in BMI^(21,23,24)^. This situation may affect emotional eating behaviours as well. This research aims to examine perceived stress and emotional eating behaviours during the COVID-19 pandemic partial quarantine based on BMI levels of healthy adults.

## Methods

### Participants

The data were collected using an online questionnaire created on Google Forms. The questionnaire was randomly distributed among the individuals using social media channels such as Instagram, Twitter and WhatsApp and via e-mail. Participants informed about the purpose of the research to adults in accordance with the inclusion criteria. Individuals between the ages of 20 and 65 who were partially quarantined (in quarantine all weekend for at least 3 months) due to COVID-19 were included in the study. Individuals from thirty-one different cities participated in the study. Seven participants were not included in the study because they were under the age of 18. The online survey data were collected between June and September 2020.

The sample size was calculated by G * power analysis (based on 95 % confidence and 80 % power) according to the report by the American Psychological Association on Stress and Nutrition. This report noted that 38 % of adults developed stress-related binge eating or unhealthy eating behaviour^(43)^. Assuming this rate to be 60 % in our study, it was determined that at least 282 people should be included in the study.

### Data collection

The questionnaire consists of four parts and a total of forty questions. The first part includes eleven questions about demographic variables such as age, weight, height, gender, education level, employment status and smoking status. The second part consists of eleven questions that examine nutritional behaviours such as mealtime, food and beverage consumption and changes in meal preparation during quarantine. Food consumption was measured with the question: ‘Which foods did you consume more after COVID-19?’. This question had several responses including whole grains (e.g. whole wheat bread, rye, oats), vegetable and fruits, nuts, meat, fish, dairy, processed foods, milky deserts, chocolate, biscuits and chips that they could chose multiple options. We asked a similar question for beverage consumption. Multiple response options included water, tea, coffee, carbonated drinks, fresh fruit juice, fruit juice, herbal tea, soda water, ayran, kefir and turnip juice. We questioned the tendency of unhealthy food consumption during the partial quarantine period with the following question: ‘Did your consumption of unhealthy foods such as chips, cookies, cakes, sugary cereals and fast food change during your stay at home?’ Participants were categorised as having a tendency to unhealthy foods if they responded, ‘I increased unhealthy food consumption’. The third part includes the Perceived Stress Scale-14 (PSS-14) and the last part includes the Emotional Eater Questionnaire (EEQ). Body weight, height and change in body weight during the partial quarantine period were collected based on the declaration. BMI was calculated using the weight and height obtained from the survey. BMI classification is: <18·5 kg/m^2^ underweight, 18·5–24·9 kg/m^2^ normal, 25·0–29·9 kg/m^2^ overweight, >30·0 kg/m^2^ obesity^(44)^.

#### Perceived Stress Scale

PSS-14 consists of fourteen items aimed at determining the perceived stress levels of individuals. PSS-14 is a scale developed by Cohen *et al.*^(45)^, which has been validated in Turkish for use in individuals over the age of 18^(46)^, and is widely applied to determine perceived helplessness and self-efficacy. This scale is prepared in a 5-point Likert type (0 no, 1, 2, 3, 4 very often), where three items are scored negatively (items 4, 5, 6). The scale is evaluated on the total score (0–32) and a high total score means higher perceived stress level^(45,46)^.

#### Emotional Eater Questionnaire

EEQ consists of ten items and three sub-dimensions. The three sub-dimensions of the EEQ are entitled as: (1) not being able to prevent eating desire-disinhibition (items 4, 5, 6, 8, 9, 10), (2) types of food type of food (items 2, 3) and (3) feeling guilty-guilt (items 1, 7). The EEQ is scored with a 4-point Likert scale (‘0’ Never, ‘1’ Sometimes, ‘2’ is generally answered and ‘3’ is always). The EEQ was used to assess emotional eating behaviour in individuals. The questionnaire developed by Garaulet *et al.*^(47)^ has been proven to be valid in Turkish for individuals over 20 years of age^(48)^. With the highest score obtained from the scale is ‘30’, higher scores indicate higher emotional eating behaviours^(47,48)^.

### Statistical analyses

The data collected were evaluated using the SPSS 25 statistics software (IBM). Data were tested to investigate whether they were normally dispersed using visual (probability plots and histograms) and analytical methods (Kolmogorov–Smirnov/Shapiro–Wilk test). Demographic variables and eating habits analysed with Pearson *χ*^2^ and independent *t*-tests. Pearson correlation coefficients were calculated to examine the interaction between BMI, EEQ and PSS-14. After checking the assumptions of the multiple linear regression analysis (linearity, covariance, independence and normality) to ensure its fit, a multiple linear regression analysis model was run to determine whether weight gain during the partial quarantine period was associated with EEQ, PSS-14, overeating and unhealthy food consumption during this period. Model fit was determined using appropriate residual and goodness of fit statistics. *P* < 0·05 was accepted as statistically significant.

## Results

Descriptive characteristics of the participants are presented in Table 1. A total of 506 participants, including 119 men (mean age: 38·59 ± 11·75 years, mean BMI: 27·28 ± 3·76 kg/m^2^) and 387 women (mean age: 30·64 ± 10·75 years, mean BMI: 23·28 ± 4·12 kg/m^2^) volunteered to participate in the study. Mean weight gain during COVID-19 was reported as 1·20 ± 1·70 kg in men and 0·91 ± 1·40 kg in women. Investigating the weight gain according to BMI, it was founded that obese participants had more weight gain than normal and overweight participants (1·76 ± 2·06 kg *v*. 1·14 ± 1·52 kg and 0·88 ± 1·48 kg in men; *P* = 0·006, 1·47 ± 1·97 kg *v*. 0·83 ± 1·27 kg and 1·10 ± 1·53 kg in women; *P* = 0·004, respectively). The EEQ and PSS-14 scores were indicated that women had significantly higher scores than men (men *v*. women; mean EEQ: 9·39 ± 5·37 *v*. 11·17 ± 5·85; mean PSS-14: 24·67 ± 8·32 *v*. 27·99 ± 7·34).

**Table 1:** Descriptive characteristics of the participants

	Men (*n* 119)		Women (*n* 387)		*P*
	Mean	SD	Mean	SD	
Age (year)	38·59	11·75	30·64	10·75	0·001**,†
Weight (kg)	85·39	12·79	62·99	12·20	0·001**,†
Weight gain					
* *Yes					
* n*	47		152		0·966‡
* *%	39·5 %		39·3 %		
* *No					
* n*	72		235		
* *%	60·5 %		60·7 %		
Weight gain during COVID-19 (kg)	1·20	1·70	0·91	1·40	0·089†
Height (cm)	1·77	0·07	1·65	0·07	0·001**,†
BMI (kg/m^2^)	27·28	3·76	23·28	4·12	0·001**,†
Changing meal times during quarantine					
* *Yes					
* n*	36		169		0·009*,‡
* *%	30·3 %		43·7 %		
* *No					
* n*	83		218		
* *%	69·7 %		56·3 %		
Increasing time spent in the kitchen during quarantine					
* *Yes					
* n*	61		302		0·001**,‡
* *%	51·3 %		78·0 %		
* *No					
* n*	58		85		
* *%	48·7 %		22·0 %		
EEQ	9·39	5·37	11·17	5·85	0·001**,†
PSS-14	24·67	8·32	27·99	7·34	0·001**,†

* *P* < 0·05.

† Independent-*t*-test.

‡ Pearson chi-square test.

** *P* < 0·001.

Table 2 shows the EEQ and PSS-14 scores according to BMI. The findings revealed that participants with higher BMI had higher EEQ and lower PSS-14 scores. Correlation analysis between BMI results, EEQ and PSS-14 scores revealed that BMI was positively correlated EEQ (*r* 0·205, *P* = 0·001) and negatively correlated with PSS-14 (*r* -0·125, *P* = 0·001) (Table 3).

**Table 2:** Perceived stress and emotional eating score change according to BMI

	BMI < 25·00 kg/m^2^ (*n* 303)		BMI > 25·00 kg/m^2^ (*n* 203)		*P*
	Mean	SD	Mean	SD	
EEQ	10·27	5·83	11·48	5·66	0·021*,†
PSS-14	28·35	8·20	25·50	7·38	0·001**,†

EEQ, Emotional Eater Questionnaire; PSS-14: Perceived Stress Scale 14.

* *P* < 0·05.

† Independent *t* test.

** *P*< 0·001.

**Table 3:** Pearson correlation analyses of EEQ and PSS-14 scores with BMI

	BMI (kg/m^2^)	
	*r*	*P*
EEQ	0·205	0·001*
PSS-14	−0·125	0·001*

EEQ, Emotional Eater Questionnaire; PSS-14, Perceived Stress Scale 14.

* *P* < 0·05.

**Fig. 1:**
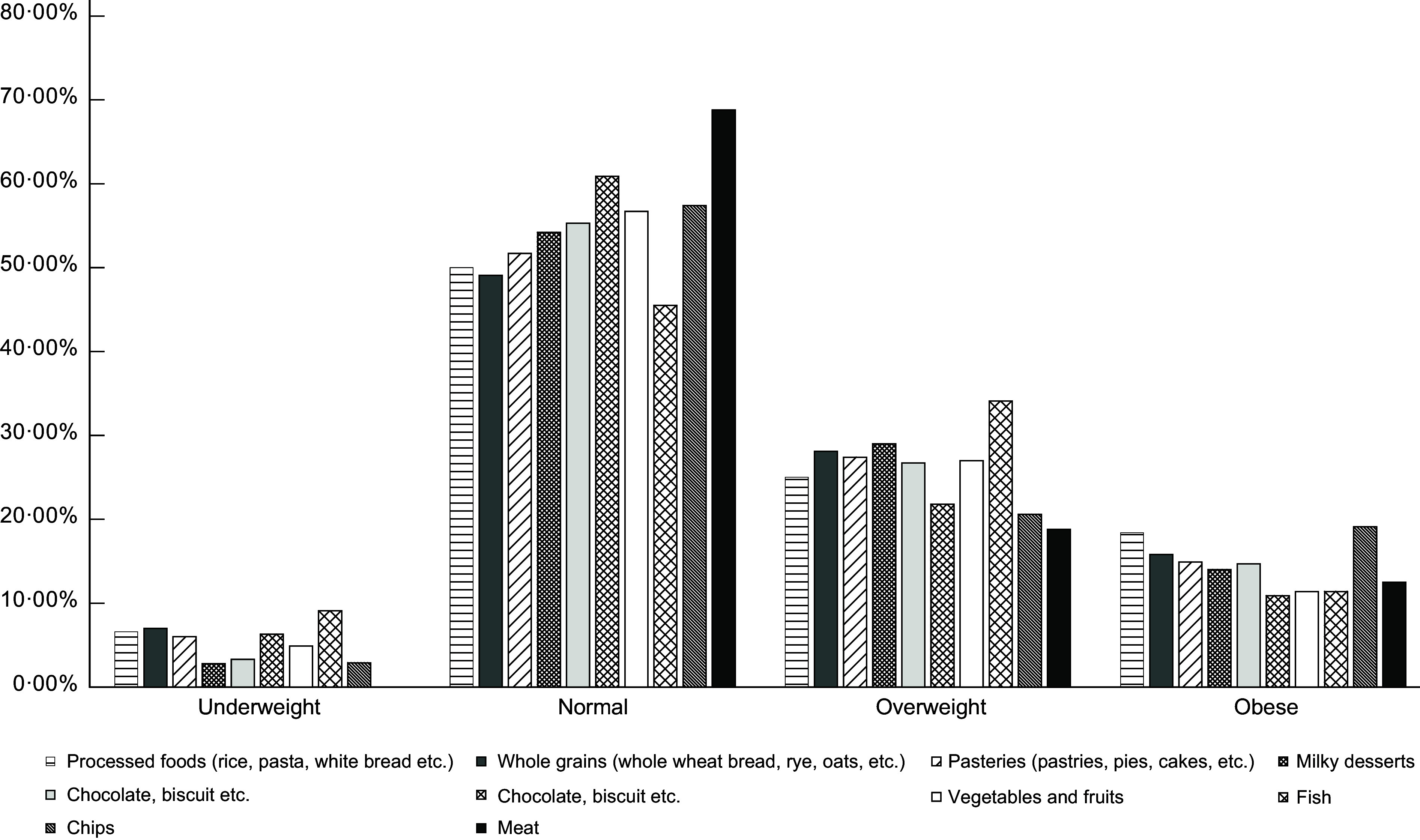
Food consumption according to BMI during COVID-19

Food consumption according to the BMI during the COVID-19 pandemic is represented in Fig. 1. Data showed that people mostly preferred to consume vegetables and fruits (56·1 %), pastries (42·9 %) and nuts (37·1 %) in all BMI groups.

**Fig. 2:**
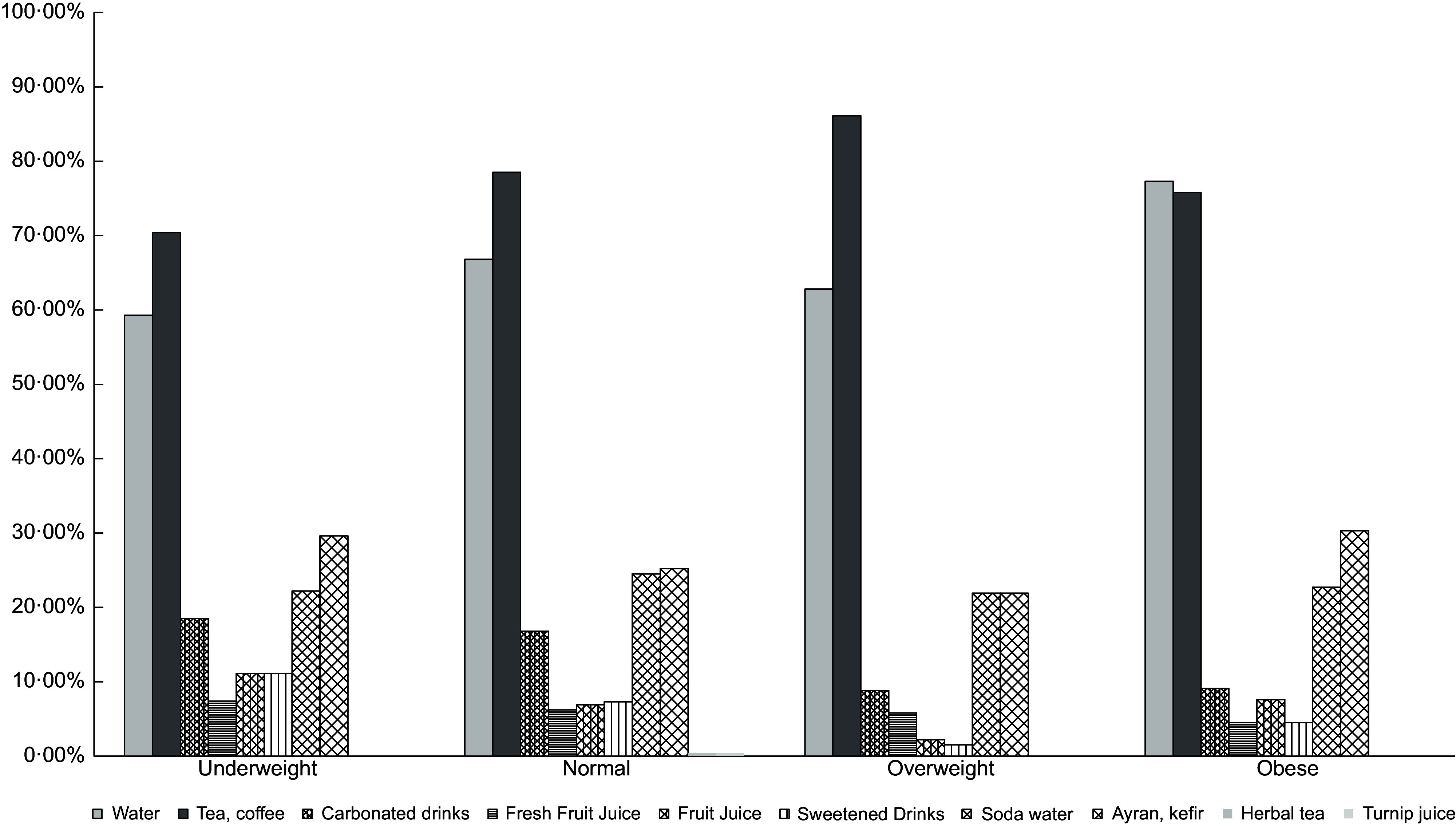
Beverage consumption according to BMI during the COVID-19

Beverage consumption according to BMI during COVID-19 is shown in Fig. 2. Water (in underweight: 59·30 %, in normal: 66·80 %, in overweight: 62·80 %, in obese: 77·30%), tea and coffee were found to be the most preferred drinks in all BMI groups (in underweight: 70·40 %, in normal: 78·50 %, in overweight: 86·10 %, in obese: 75·80 %). While consumption of tea, coffee, fresh fruit juice and carbonated beverages increased, consumption of water, ayran, kefir, fruit juice and sugary beverage decreased in underweight participants. In overweight individuals, while the consumption of water, carbonated drinks, fresh fruit juice, fruit juice, sweetened drinks, soda and ayran increased, tea and coffee consumption decreased. The consumption of sweetened and carbonated drinks increased twice in obese participants in those compared with other BMI groups.

We ran a regression analysis to identify whether weight gain during the partial quarantine period could be predicted using data on EEQ, PSS-14, overeating and unhealthy food choice during this period (Table 4). The regression model showed that 29 % of weight gain during the partial quarantine period could be explained by emotional eating, eating more while at home and unhealthy eating behaviours at home. In contrast, perceived stress was not associated with weight gain.

**Table 4:** Multiple linear regression analyses of the variables associated with weight gain during COVID-19

Variable	Weight gain during COVID-19			
	*β*	*P*	95 % CI	
PSS-14	0·054	0·207	−0·006	0·026
EEQ	0·173	0·001*	0·001	0·015
Eating more while at home	0·171	0·001*	0·338	0·505
Consuming unhealthy foods while at home	0·224	0·001*	0·037	0·199

PSS-14, Perceived Stress Scale-14; EEQ, Emotional Eater Questionnaire.

* *P* < 0·001.

## Discussion

In this paper, we present the changes related to eating behaviours, weight-gain patterns and perceived stress according to BMI during the COVID-19 pandemic lockdown. Our main findings were that BMI was positively associated with EEQ scores, suggesting that people with higher BMI are more prone to emotional eating. Additionally, weight gain during quarantine was associated with increased eating tendency and consumption of unhealthy foods at home. However, the hypothesis that people with higher BMI during quarantine would have higher stress levels was rejected due to findings suggesting that BMI was negatively correlated with PSS-14 scores.

We observed a positive correlation between BMI and EEQ scores. Several studies have reported that obese individuals tend to eat emotionally and BMI is positively associated with EEQ scores^(27,34,49–52)^. The researchers argued that certain strong emotions, such as anxiety, restlessness, anger, fear, joy and sadness, can cause significant changes in eating behaviours by increasing the motivation to eat and the amount of food consumed, and changing food choices towards unhealthy foods. People with emotional eating symptoms often react to overwhelming conditions by increasing their food intake and altering food choices with high in fat and sugar, while others do not alter or reduce their food consumption in these situations. Increased food consumption can also increase the tendency to binge-eating^(29–31,33–35,42,43,50,53–57)^. Because we know that COVID-19 creates enormous stress and pressure in our lives and changes almost the entire lifestyle, it is inevitable to affect eating behaviours, especially for individuals who already tend to eat a lot. For this reason, obese people, especially those who notice an increase in food consumption or emotional eating attacks during the COVID-19 quarantine, should consider seeking professional support to endure this negative and uncertain process and prevent unwanted weight gain.

Consistent with other COVID-19 studies, weight gain during home quarantine was observed in both men and women (39·5 and 39·3 %, respectively). Men reported an average weight gain of 1·20 kg, whereas women stated about 0·91 kg of increase in weight. A study by Górnicka *et al.*^(58)^ showed that staying at home during COVID-19 decreased physical activity levels and resulted in undesirable eating behaviours such as snacking and overeating, thus causing a disruption in energy balance and weight gain. Another study conducted in Obesity Unit examined the weight changes and BMI before and after COVID-19 lockdown^(8)^. They found that after the COVID-19 lockdown, weight gain and BMI increased by about +1·51 kg and + 0·58 kg/m^2^, respectively^(8)^. It was stated that 43·7 % of women and 30·3 % of men were changed their daily eating habits and the time spent in the kitchen increased. Additionally, Di Renzo *et al.*^(10)^ investigated the lockdown impact on eating habits and lifestyle changes throughout four different parts of Italy. Researchers stated that 48·6 % of the participants reported weight gain. The frequency and habits of meals changed and the time they spent in the kitchen increased in 57·8 % of the participants However, unlike other studies, no change was observed in the mealtime in our study^(10)^. This is probably due to differences in lockdown. While several countries such as Italy applied a full-time lockdown strategy, the lockdown in Turkey was limited by weekends. Therefore, it is an expectable situation that there is no change in mealtime as people continue their normal work schedule on weekdays.

We found that PSS-14 scores negatively correlated with BMI. This result is probably due to the different stress responses of individuals^(59)^. Although the stress response varies according to the severity and duration of the stress, it can be quite challenging^(60)^. While mild stress creates positive results by increasing the attention of individuals^(61)^, chronic stress can lead to serious physiological and psychological consequences^(60,62)^. It is very frustrating not knowing when the COVID pandemic will end and what the definitive treatment is, which can lead to chronic stress in individuals. In addition, although it is well known that stress can affect eating habits, how it affects it is controversial. Some people respond to high-stress situations by reducing their food intake, while others increase their food consumption to suppress undesirable emotions^(30,53)^. The fact that perceived stress was higher in participants with low BMI suggests that COVID stress may also lead to shut down appetite.

In this study, EEQ scores were positively correlated with PSS-14 scores (*r* 0·275 in males, *r* 0·279 in females) regardless of BMI levels (*P* < 0·001). With the prolonged COVID-19 outbreak, people feel more stressed out of an increased fear of infection, the loss of loved ones or financial loss^(15–17,19,63,64)^. Increased stress can change food and beverage consumption in individuals. A longitudinal study evaluating the effect of natural disasters on emotional eating stated that the tendency of unhealthy foods increases during stressful periods^(55)^. In the present study, vegetables, fruits and pastries were found to be mostly preferred foods. While vegetables and fruits are considered beneficial foods with their specialties rich in phytochemicals, vitamins, minerals and fibre for maintaining health and boosting the immune system during the pandemic, increased consumption of pastries can create undesirable consequences such as weight gain. Further, sweetened beverage consumption has doubled in obese individuals. These findings show that eating habits change towards unhealthy foods, especially in obese patients, during COVID-19 pandemic. This finding is supported by another study that showed that during the COVID-19 outbreak, the consumption of sweetened and carbonated drink decreased while the consumption of homemade desserts, pizza and bread increased^(8)^. Cohort studies in the Netherlands, Finland, France, USA and Korea reported that weight gain as a consequence of emotional eating is a crucial risk factor for obesity^(65–69)^. Therefore, there is an urgent need to develop some public health strategies to reduce obesity risks, attenuate perceived stress and improve well-being during COVID-19.

In good agreement with several studies, the regression analyses of this study indicated that increased weight gain during the COVID-19 lockdown significantly increased the risk of emotional eating behaviours, including eating more at home and consuming unhealthy foods at home. However, no association was found between weight gain and perceived stress during COVID-19. One possible explanation is that stress can cause hypophagia that restricts eating behaviour for some people.

This study had some limitations. First, we were unable to evaluate food and beverage consumption prior to the COVID-19 outbreak. Second, emotional eating and perceived stress were studied using self-reported survey data. Third, although we tried to reach more male participants, sex distribution is not balanced. In addition, anthropometric measurements and weight gain were taken according to the declaration. On the other hand, the strength of this study is that, to our knowledge, this is the first study to evaluate the interaction of emotional eating and perceived stress to BMI during the COVID-19 pandemic. With the study, we highlighted that the COVID-19 lockdown does not only affect physical health but also influences psychological well-being, which can pose a greater burden on public health, such as increased obesity and obesity-related disorders.

## Conclusion

Our results suggest that staying home during the COVID-19 pandemic may cause emotional eating, snacking and overeating, and therefore lead to weight gain. Additionally, individuals with higher BMI have been associated with a higher propensity to emotional eating. For this reason, the change in eating behaviour and the increase in the time spent at home may create a huge burden, especially for obese individuals.

The lockdown is a good precaution to stop the spread of the virus, but there are some health risks associated with physical inactivity, weight gain, behavioural changes and social isolation. Further studies should focus on the impact of the COVID-19 outbreak on eating behaviour and perceived stress. As we know that the lockdown process will continue for a while, some public health strategies focusing on healthy eating and physical activity at home should be developed to eliminate the health risks associated with increased obesity during the lockdown.

## References

[1] Huang C , Wang Y , Li X et al. (2020) Clinical features of patients infected with 2019 novel coronavirus in Wuhan, China. Lancet 395, 497–506. 31986264 10.1016/S0140-6736(20)30183-5 PMC7159299

[2] Johnson M (2020) Wuhan 2019 novel coronavirus – 2019-nCoV. Mater Methods 10, 1–5.

[3] REPUBLIC OF TURKEY MINISTRY OF HEALTH COVID-19 INFORMATION PAGE, 2 April 20, 25. https://covid19.saglik.gov.tr/TR-66301/covid-19-rehberi.html (accessed July 2021).

[4] Republic of Turkey Ministry of Health (2021) COVID-19 Information. Turkey COVID-19 Patient Table. https://covid19.saglik.gov.tr/TR-66935/genel-koronavirus-tablosu.html (accessed July 2021).

[5] Pitlik SD (2020) Covid-19 compared to other pandemic diseases. Rambam Maimonides Med J 11, 1–17. 32792043 10.5041/RMMJ.10418 PMC7426550

[6] World Health Organization (WHO) *COVID-19: Physical Distancing*. 2020. https://www.who.int/westernpacific/emergencies/covid-19/information/physical-distancing (accessed July 2021).

[7] Republic of Turkey Ministry of Health (2020) COVID-19 Pandemic Managment and Work Guide, 1–459. https://covid19.saglik.gov.tr/TR-66393/covid-19-salgin-yonetimi-ve-calisma-rehberi.html (accessed July 2021).

[8] Pellegrini M , Ponzo V , Rosato R et al. (2020) Changes in weight and nutritional habits in adults with obesity during the “lockdown” period caused by the COVID-19 virus emergency. Nutrients 12, 1–11. 10.3390/nu12072016 PMC7400808 32645970

[9] Ryan DH , Ravussin E & Heymsfield S (2020) COVID 19 and the patient with obesity – the editors speak out. Obesity 28, 847. 32237212 10.1002/oby.22808 PMC7228389

[10] Di Renzo L , Gualtieri P , Pivari F et al. (2020) Eating habits and lifestyle changes during COVID-19 lockdown: an Italian survey. J Transl Med 18, 1–15. 32513197 10.1186/s12967-020-02399-5 PMC7278251

[11] Banerjee S & Samaddar B (2020) Impact of COVID-19 lockdown on overweight typically managed by easy diet planning – a mini review. Int J Tech 10, 39–42.

[12] Lippi G , Henry BM , Bovo C et al. (2020) Health risks and potential remedies during prolonged lockdowns for coronavirus disease 2019 (COVID-19). Diagnosis 7, 85–90. 32267243 10.1515/dx-2020-0041

[13] Matsungo TM & Chopera P (2020) The effect of the COVID-19 induced lockdown on nutrition, health and lifestyle patterns among adults in Zimbabwe. BMJ Nutrition Prevention & Health, 1–8. 10.1136/bmjnph-2020-000124 PMC7841831 33521530

[14] Sandhu K , Kaur B & Author C-A (2020) Impact of COVID-19 lockdown on the dietary pattern and physical activity of people article history. Horiz J Hum Soc Sci 2, 205–216.

[15] Sønderskov KM , Dinesen PT , Santini ZI et al. (2020) The depressive state of Denmark during the COVID-19 pandemic. Acta Neuropsychiatr 32, 17–19. 10.1017/neu.2020.15 PMC7176490 32319879

[16] Huang Y & Zhao N (2020) Generalized anxiety disorder, depressive symptoms and sleep quality during COVID-19 outbreak in China: a web-based cross-sectional survey. Psychiatry Res 288, 112954. 32325383 10.1016/j.psychres.2020.112954 PMC7152913

[17] Liang L , Ren H , Cao R et al. (2020) The effect of COVID-19 on youth mental health. Psychiatr Q 91, 3–5. 10.1007/s11126-020-09744-3 PMC7173777 32319041

[18] Cao W , Fang Z , Hou G et al. (2020) The psychological impact of the COVID-19 epidemic on college students in China. Psychiatry Res 287, 112934. 32229390 10.1016/j.psychres.2020.112934 PMC7102633

[19] Serafini G , Parmigiani B , Amerio A et al. (2020) The psychological impact of COVID-19 on the mental health in the general population. QJM 113, 229–235. 32569360 10.1093/qjmed/hcaa201 PMC7337855

[20] Moccia L , Janiri D , Pepe M et al. (2020) Affective temperament, attachment style, and the psychological impact of the COVID-19 outbreak: an early report on the Italian general population. Brain Behav Immun 87, 75–79. 32325098 10.1016/j.bbi.2020.04.048 PMC7169930

[21] Shen W , Long LM , Shih CH et al. (2020) A humanities-based explanation for the effects of emotional eating and perceived stress on food choice motives during the COVID-19 pandemic. Nutrients 12, 1–18. 10.3390/nu12092712 PMC7551550 32899861

[22] Elmacloǧlu F , Emiroǧlu E , Ülker MT et al. (2021) Evaluation of nutritional behaviour related to COVID-19. Public Health Nutr 24, 512–518. 33070798 10.1017/S1368980020004140 PMC7737137

[23] Cecchetto C , Aiello M , Gentili C et al. (2021) Increased emotional eating during COVID-19 associated with lockdown, psychological and social distress. Appetite 160, 105122. 33453336 10.1016/j.appet.2021.105122 PMC9755826

[24] Al-Musharaf S (2020) Prevalence and predictors of emotional eating among healthy young Saudi women during the COVID-19 pandemic. Nutrients 12, 1–17. 10.3390/nu12102923 PMC7598723 32987773

[25] Serin Y (2018) Emotional eating, the factors which affect food intake and basic approaches of nursing care. J Psychiatr Nurs 9, 135–146.

[26] Van Strien T , Cebolla A , Etchemendy E et al. (2013) Emotional eating and food intake after sadness and joy. Appetite 66, 20–25. 23470231 10.1016/j.appet.2013.02.016

[27] van Strien T , Herman CP & Verheijden MW (2009) Eating style, overeating, and overweight in a representative Dutch sample. Does external eating play a role? Appetite 52, 380–387. 19100301 10.1016/j.appet.2008.11.010

[28] Yamamoto K , Okazaki A & Ohmori S (2011) The relationship between psychosocial stress, age, BMI, CRP, lifestyle, and the metabolic syndrome in apparently healthy subjects. J Physiol Anthropol 30, 15–22. 21307616 10.2114/jpa2.30.15

[29] Järvelä-Reijonen E , Karhunen L , Sairanen E et al. (2016) High perceived stress is associated with unfavorable eating behavior in overweight and obese Finns of working age. Appetite 103, 249–258. 27108837 10.1016/j.appet.2016.04.023

[30] Tan CC & Chow CM (2014) Stress and emotional eating: the mediating role of eating dysregulation. Pers Individ Differ 66, 1–4.

[31] Torres SJ & Nowson CA (2007) Relationship between stress, eating behavior, and obesity. Nutrition 23, 887–894. 17869482 10.1016/j.nut.2007.08.008

[32] Konttinen H (2012) Dietary habits and obesity: the role of emotional and cognitive factors. https://core.ac.uk/download/pdf/14922772.pdf (accessed July 2021).

[33] Konttinen H (2020) Emotional eating and obesity in adults: the role of depression, sleep and genes. Proc Nutr Soc 79, 283–289. 32213213 10.1017/S0029665120000166

[34] Lazarevich I , Irigoyen Camacho ME , Velázquez-Alva MdC et al. (2016) Relationship among obesity, depression, and emotional eating in young adults. Appetite 107, 639–644. 27620648 10.1016/j.appet.2016.09.011

[35] Conner M , Fitter M & Fletcher W (1999) Stress and snacking: a diary study of daily hassles and between-meal snacking. Psychol Health 14, 51–63.

[36] Bray GA (2013) Energy and fructose from beverages sweetened with sugar or high-fructose corn syrup pose a health risk for some people. Adv Nutr 4, 220–225. 23493538 10.3945/an.112.002816 PMC3649102

[37] Manna P & Jain SK (2015) Obesity, oxidative stress, adipose tissue dysfunction, and the associated health risks: causes and therapeutic strategies. Metab Syndr Relat Disord 13, 423–444. 26569333 10.1089/met.2015.0095 PMC4808277

[38] Stanhope KL (2016) Sugar consumption, metabolic disease and obesity: the state of the controversy. Crit Rev Clin Lab Sci 53, 52–67. 26376619 10.3109/10408363.2015.1084990 PMC4822166

[39] Jayne JM , Ayala R , Karl JP et al. (2020) Body weight status, perceived stress, and emotional eating among US army soldiers: a mediator model. Eat Behav 36, 101367. 32018191 10.1016/j.eatbeh.2020.101367

[40] Roberts C , Troop N , Connan F et al. (2007) The effects of stress on body weight: biological and psychological predictors of change in BMI. Obesity 15, 3045–3055. 18198314 10.1038/oby.2007.363

[41] Lingswiler VM , Crowther JH & Stephens MAP (1989) Emotional and somatic consequences of binge episodes. Addict Behav 14, 503–511. 2589128 10.1016/0306-4603(89)90070-1

[42] Richardson AS , Arsenault JE , Cates SC et al. (2015) Perceived stress, unhealthy eating behaviors, and severe obesity in low-income women. Nutr J 14, 1–10. 26630944 10.1186/s12937-015-0110-4 PMC4668704

[43] American Psychological Association (2013) *Stress and Eating*. https://www.apa.org/news/press/releases/stress/2013/eating (accessed July 2021).

[44] World Health Organization (WHO) *Body Mass Index – BMI*. 2021. https://www.euro.who.int/en/health-topics/disease-prevention/nutrition/a-healthy-lifestyle/body-mass-index-bmi (accessed July 2021).

[45] Cohen S , Kamarck T & Mermelstein R (1983) A global measure of perceived stress. J Health Soc Behav 24, 385–396. 6668417

[46] Eskin M ; Harlak H , Demirkıran F et al. (2013) The adaptation of the perceived stress scale into Turkish: a reliability and validity analysis. Yeni Symp 51, 132–140.

[47] Garaulet M , Canteras M , Morales E et al. (2012) Validation of a questionnaire on emotional eating for use in cases of obesity; the Emotional Eater Questionnaire (EEQ) Nutr Hosp 27, 645–651. 22732995 10.1590/S0212-16112012000200043

[48] Arslantaş H , Dereboy F , Yüksel R et al. (2019) Validity and reliability of the Turkish version of the Emotional Eater Questionnaire (EEQ-TR) Türk Psikiyatr Derg 30, 1–10. 10.5080/u23520 32594500

[49] Nolan LJ , Halperin LB & Geliebter A (2010) Emotional appetite questionnaire. construct validity and relationship with BMI. Appetite 54, 314–319. 20005275 10.1016/j.appet.2009.12.004 PMC3835339

[50] Diggins A , Woods-Giscombe C & Waters S (2015) The association of perceived stress, contextualized stress, and emotional eating with body mass index in college-aged Black women. Eat Behav 19, 188–192. 26496005 10.1016/j.eatbeh.2015.09.006

[51] Konttinen H , Silventoinen K , Sarlio-Lähteenkorva S et al. (2010) Emotional eating and physical activity self-efficacy as pathways in the association between depressive symptoms and adiposity indicators. Am J Clin Nutr 92, 1031–1039. 20861176 10.3945/ajcn.2010.29732

[52] Péneau S , Ménard E , Méjean C et al. (2013) Sex and dieting modify the association between emotional eating and weight status. Am J Clin Nutr 97, 1307–1313. 23576047 10.3945/ajcn.112.054916

[53] Leigh Gibson E (2006) Emotional influences on food choice: sensory, physiological and psychological pathways. Physiol Behav 89, 53–61. 16545403 10.1016/j.physbeh.2006.01.024

[54] Allison KC , Lundgren JD , Reardon JPO et al. (2008) The night eating questionnaire (NEQ): psychometric properties of a measure of severity of the night eating syndrome. Eat Behav 9, 62–72. 18167324 10.1016/j.eatbeh.2007.03.007

[55] Kuijer RG & Boyce JA (2012) Emotional eating and its effect on eating behaviour after a natural disaster. Appetite 58, 936–939. 22369959 10.1016/j.appet.2012.02.046

[56] Sproesser G , Schupp HT & Renner B (2014) The bright side of stress-induced eating: eating more when stressed but less when pleased. Psychol Sci 25, 58–65. 24166853 10.1177/0956797613494849

[57] Sproesser G , Schupp HT & Renner B (2014) The bright side of stress-induced eating: eating more when stressed but less when pleased. Psychol Sci 25, 58–65. 24166853 10.1177/0956797613494849

[58] Górnicka M , Drywień ME , Zielinska MA et al. (2020) Dietary and lifestyle changes during covid-19 and the subsequent lockdowns among polish adults: a cross-sectional online survey plifecovid-19 study. Nutrients 12, 1–23. 10.3390/nu12082324 PMC7468840 32756458

[59] Yau YHC & Potenza MN (2013) Stress and eating behaviors. Minerva Endocrinol 38, 255–267. 24126546 PMC4214609

[60] McEwen BS (2004) Protection and damage from acute and chronic stress: allostasis and allostatic overload and relevance to the pathophysiology of psychiatric disorders. Ann N Y Acad Sci 1032, 1–7. 15677391 10.1196/annals.1314.001

[61] Selye H (1976) The Stress of Life. New York: McGraw-Hill.

[62] Pasquali R (2012) The hypothalamic-pituitary-adrenal axis and sex hormones in chronic stress and obesity: pathophysiological and clinical aspects. Ann N Y Acad Sci 1264, 20–35. 22612409 10.1111/j.1749-6632.2012.06569.x PMC3464358

[63] CSTS (2020) Psychological effects of quarantine during the coronavirus outbreak: what healthcare providers need to know. https://www.cstsonline.org/assets/media/documents/CSTS_FS_Psychological_Effects_Quarantine_During_Coronavirus_Outbreak_Providers.pdf (accessed July 2021).

[64] Cullen W , Gulati G & Kelly BD (2020) Mental health in the COVID-19 pandemic. QJM 113, 311–312. 32227218 10.1093/qjmed/hcaa110 PMC7184387

[65] Koenders PG & Van Strien T (2011) Emotional eating, rather than lifestyle behavior, drives weight gain in a prospective study in 1562 employees. J Occup Environ Med 53, 1287–1293. 22027541 10.1097/JOM.0b013e31823078a2

[66] Song YM , Lee K , Sung J et al. (2013) Changes in eating behaviors and body weight in Koreans: the healthy twin study. Nutrition 29, 66–70. 22858202 10.1016/j.nut.2012.03.014

[67] van Strien T , Konttinen H , Homberg JR et al. (2016) Emotional eating as a mediator between depression and weight gain. Appetite 100, 216–224. 26911261 10.1016/j.appet.2016.02.034

[68] Bénard M , Bellisle F , Etilé F et al. (2018) Impulsivity and consideration of future consequences as moderators of the association between emotional eating and body weight status. Int J Behav Nutr Phys Act 15, 1–11. 30189878 10.1186/s12966-018-0721-1 PMC6127957

[69] Vittengl JR (2018) Mediation of the bidirectional relations between obesity and depression among women. Psychiatry Res 264, 254–259. 29655968 10.1016/j.psychres.2018.03.023

